# Impact of biochar on enzyme activity, respiration, and microbial population in irrigated farmland

**DOI:** 10.3389/fmicb.2026.1924861

**Published:** 2026-09-07

**Authors:** Junfei Tong, Lining Zhao, Lili Su, Ruxue Li, Xiaohua Ye, Ningju Xi, Shufang Zhang, Xayda Mamat, Qihua Zhu, Yuxin Chen, Weijun Yang

**Affiliations:** 1 College of Agronomy, Xinjiang Agricultural University, Urumqi, China; 2 National Forest and Grass Germplasm Resources Facilities Preservation Library Xinjiang Branch, Xinjiang Academy of Forestry Sciences, Urumqi, China

**Keywords:** biochar, CO_2_ emissions, nitrogen fertilization, soil enzyme activity, soil microorganisms

## Abstract

**Purpose:**

This study investigated the effects of different biochar application rates on wheat yield, soil microbial activity, enzyme function, and CO_2_ emissions under two nitrogen fertilization levels, aiming to identify an optimal strategy for improving soil health and reducing emissions.

**Methods:**

A randomized block design was used with eight treatments combining two nitrogen rates (conventional N1: 300 kg·ha^−1^, reduced N2: 255 kg·ha^−1^) and four biochar doses (0, 10, 20, 30 t·ha^−1^). Soil microbial community, respiration, enzyme activities, and CO_2_ emissions were analyzed.

**Results:**

Biochar enhanced overall soil microbial activity and diversity. The highest microbial activity indices occurred with conventional nitrogen and moderate biochar (N1B2). All biochar treatments increased microbial diversity, with ester consumption as the primary carbon source. The combination of reduced nitrogen and moderate biochar (N2B2) lowered cumulative CO_2_ emissions by 4.42% compared to reduced nitrogen alone. Both nitrogen and biochar increased sucrase, urease, and catalase activities.

**Conclusion:**

The application of reduced nitrogen (255 kg·ha^−1^) with moderate biochar (20 t·ha^−1^) is identified as an optimal strategy to enhance the soil micro environment and mitigate CO_2_ emissions in irrigated wheat fields, though its long-term effects require further assessment.

## 1 Introduction

Arid and semi-arid regions play a crucial role in global food security, yet they face intertwined challenges from climate change and the need for sustainable agricultural practices. Typical arid irrigated areas in China, like Xinjiang, exhibit low stability of soil carbon pool and limited efficiency of utilizing nitrogen fertilizers. Excessive long-term fertilization sustains crop yields, but leads to environmental issues, such as degraded soil quality and higher emission of greenhouse gases. Against the backdrop of global efforts toward carbon neutrality and sustainable agriculture, soil fertility and carbon sequestration needs to be improved synergistically through integrated soil management strategies.

Biochar application is a key strategy for enhancing soil health and carbon sequestration. Its porosity and chemical stability improve the physical safety and chemical adsorption of the organic carbon in soil, while also influencing carbon and nitrogen cycling in the soil by shaping the microbial habitats. However, the ongoing research efforts are mainly focused on the short-term implications of sole biochar application, alone. There is a limited understanding of the microbial-driven mechanisms underlying the cooperative interactions of biochar with nitrogen fertilizers under long-term fertilization. Particularly, in the arid regions with pronounced coupling of soil moisture and nutrient levels, the effects of biochar on energy-acquisition strategies of microbes, extracellular enzyme activity, and the functional evolution of microbial populations remain elusive.

In this study, a typical irrigated farmland in the northern Xinjiang was chosen for the field trials to examine the impact of biochar and nitrogen fertilizer on microbial metabolic efficiency, community composition, and the release of CO_2_. Therefore, this study aimed to evaluate the effects of different biochar application rates under conventional and reduced nitrogen fertilization on (i) the carbon-source utilization profiles and functional diversity of the culturable soil microbial fraction, (ii) soil sucrase, urease, and catalase activities, and (iii) soil respiration dynamics and cumulative total soil CO₂ emissions in irrigated wheat farmland. We further examined whether these responses were affected by biochar rate, nitrogen application rate, and their interaction. We hypothesized that biochar would alter microbial carbon-source utilization profiles and soil enzyme activities in a dose-dependent manner, and that these responses would depend on nitrogen application rate. We further hypothesized that, under reduced nitrogen input, a moderate biochar application rate would reduce cumulative total soil CO₂ emissions compared with reduced nitrogen treatment without biochar, whereas a higher biochar rate would not necessarily provide an additional reduction.

## 2 Materials and methods

### 2.1 Experimental plots

The field experiment was conducted in the Qitai Wheat Test Station in Xinjiang (89°13′ - 91°22′E; 42°25′ - 45°29′N). This site has a temperate continental climate, with a maximum temperature of 39 °C and an average temperature of 5.5 °C yearly. The average temperatures in January and July were −18.9 °C and 22.6 °C, correspondingly. On average, the annual relative humidity in the area is 60% and annual rainfall is 269.4 mm, with 153 days of frost-free season (late April to early October). The field has a sandy loam soil with following characteristics: pH 8.3, organic matter: 13.8 g·kg^−1^, total nitrogen: 2.2 g·kg^−1^, salt level: 1.4 g·kg^−1^, readily available potassium and phosphorus levels: 147.0 mg·kg^−1^ and 11.4 mg·kg^−1^, respectively, and alkaline hydrolysis nitrogen level of 128.7 mg·kg^−1^.

### 2.2 Materials

Corn-straw biochar (produced through 4 h pyrolysis at 450 °C) was utilized by Jinhefu Shenyang agricultural technology development corporation, China. The basic characteristics of this biochar were as follows: pH 9.3, total nitrogen 21.8 g·kg^−1^, and available phosphorus and nitrogen levels of 200.9 mg·kg^−1^ and 5.4 mg·kg^−1^, respectively. The local spring wheat variety, i.e., “Xinchun 37,” was used for the experiment.

### 2.3 Experimental design

According to a randomized block design, eight treatment groups were designed, with four levels of biochar application (B0: 0 kg·ha^−1^, B1: 10 × 10^3^ kg·ha^−1^, B2: 20 × 10^3^ kg·ha^−1^, and B3: 30 × 10^3^ kg·ha^−1^) and two levels of nitrogen fertilization (N1: 300 kg·ha^−1^ and N2: 255 kg·ha^−1^). Based on the biochar and nitrogen fertilization levels, the groups were named as N1B0, N1B1, N1B2, N1B3, N2B0, N2B1, N2B2, and N2B3. Each treatment group had 3 replicates. Each plot had an area of 9 m^2^. Spring wheat was sown in the field in 0.2 m equally-spaced strips at a rate of 450 × 10^4^·ha^−1^ on April 12th 2021. Before sowing, biochar and nitrogen fertilizer were manually added into the soil via tillage at 30 cm depth. No other fertilizer was applied subsequently. During the entire reproductive growth period, the experimental field was irrigated 8 times. Throughout the wheat growing season, the field was irrigated eight times, with a total irrigation amount of 4,500 m^3^ ha^−1^.

### 2.4 Sampling and measurements

#### 2.4.1 Soil sampling

During the harvesting period, soil was sampled from 30 cm layer using five-point collection method. The samples collected from a group were properly mixed to create a representative sample. After removing roots and debris, the soil samples were sieved (0.2 cm mesh) and dried in air.

#### 2.4.2 Measurements

(1) Functional diversity of soil microbes.

BIOLOG ECO well plates (Hayward, USA) were employed to examine the microbial functional diversity in soil samples. 2.0 g soil sample was mixed into 18 mL of sterilized saline solution (0.87% w·v^−1^), and then the solution was shaken for 20 min. Then, 150 μL of the soil suspension was inoculated into each well of the BIOLOG ECO plate and incubated in the dark at 25 °C. Employing a plate reader (EL808, BioTek), absorbance of the solution in each well was recorded at 590 nm for 8 days at regular intervals of 18, 44, 68, 89, 115, 138 and 144 h. Carbon-source utilization for each substrate category was calculated as the mean OD₅₉₀ per substrate within that category (total category absorbance divided by the number of substrates in the category), thereby accounting for unequal well numbers across the six carbon-source groups.

(2) Soil respiration rate of soil.

Three days prior to first analysis, polyvinyl chloride (PVC) collars, with a diameter of 10 cm and a height of 5 cm, were placed upright 5 cm deep into the soil in each plot between the crop rows. The soil surrounding the exterior of each collar was firmly pressed to stop the leakage of gas. An automated soil CO_2_ flux system (LI-8100, LI-COR Inc., USA) was used to monitor the released CO_2_ weekly from each collar between 9:00 to 11:00 a.m. during the growth period of wheat. Temperature of soil at 5 cm depth was determined by placing stem thermometers near the collar.

The grand total of the daily CO_2_ fluxes throughout the growing period of wheat represented cumulative CO_2_ emissions. To determine the daily CO_2_ flux of the unmeasured days, the average CO_2_ flux of two adjoining quantification days was multiplied by the corresponding duration. The yield-scaled CO_2_ release was calculated by dividing the cumulative CO_2_ emissions with wheat yield. Because no root-exclusion treatment was applied, the measured CO₂ flux represented total soil respiration, including both root respiration and heterotrophic microbial respiration. The relative contributions of these two components were not partitioned in this study.

(3) Soil enzyme activity.

The activities of sucrose, urease, and catalase were quantified by dinitro-salicylic acid colorimetric method, phenol-sodium hypochlorite colorimetric method, and KMnO_4_ titration, correspondingly (Yuan et al., 2017; Liu et al., 2017). The geometric mean enzyme activity (GMEA) was calculated as the geometric mean of sucrase, urease, and catalase activities.

#### 2.4.3 Calculations

(1) Soil microbial functional diversity.

During BIOLOG ECO plate test, average well color development (AWCD) denotes the carbon metabolic capacity of microbes, reflecting the microbial activity and functional diversity (Yerulker et al., 2023). AWCD was quantified employing the following Equations 1−4:


AWCD=∑(C−R)/31 (1)

Here, C signifies the absorbance of the solution in a well, while R is the absorbance of the control well.

AWCD indicates the activity of microbes in soil, while diversity indices (i.e., McIntosh, Simpson, and Shannon) reflects species distribution, as well as the functional diversity and composition of microbial community.

Mclntosh index (U), indicating the uniformity of microbial populations, was determined as specified below:


$$U=\sqrt{\sum n_{i}^{2}}$$ (2)

Simpson index (D), representing the predominant microbial community species, was determined as follows:


$$D=1-\sum p_{i}^{2}$$ (3)

Shannon index (H), reflecting species richness, was determined as follows:


$$H=-\sum p_{i}\ln p$$ (4)

Where *P* is the ratio of the absorbance in i well to the total absorbance.

(2) Cumulative emission of CO_2_.

M = ∑(F_i + 1_ + Fi)/2 × (t_i + 1_-ti) × 24(5)

Here M is the combined CO_2_ flux emitted from soil, F is the CO_2_ flux emitted from the soil, t is the sampling day, and I is the total number of samples.

### 2.5 Statistical analysis

Statistical analyses were performed using DPS 7.05. The experiment followed a balanced 2 × 4 factorial arrangement within a randomized block design, with two nitrogen application rates and four biochar application rates. Each treatment combination included three independent field replicates. The effects of nitrogen rate, biochar rate, and their interaction were evaluated using two-way analysis of variance (ANOVA). When significant differences were detected, treatment means were compared using Duncan’s multiple range test at *p* < 0.05. Origin 2021 was used to prepare the figures.

## 3 Results

### 3.1 Effect of biochar dose on carbon metabolic activity of soil microbes

**Figure 1:**
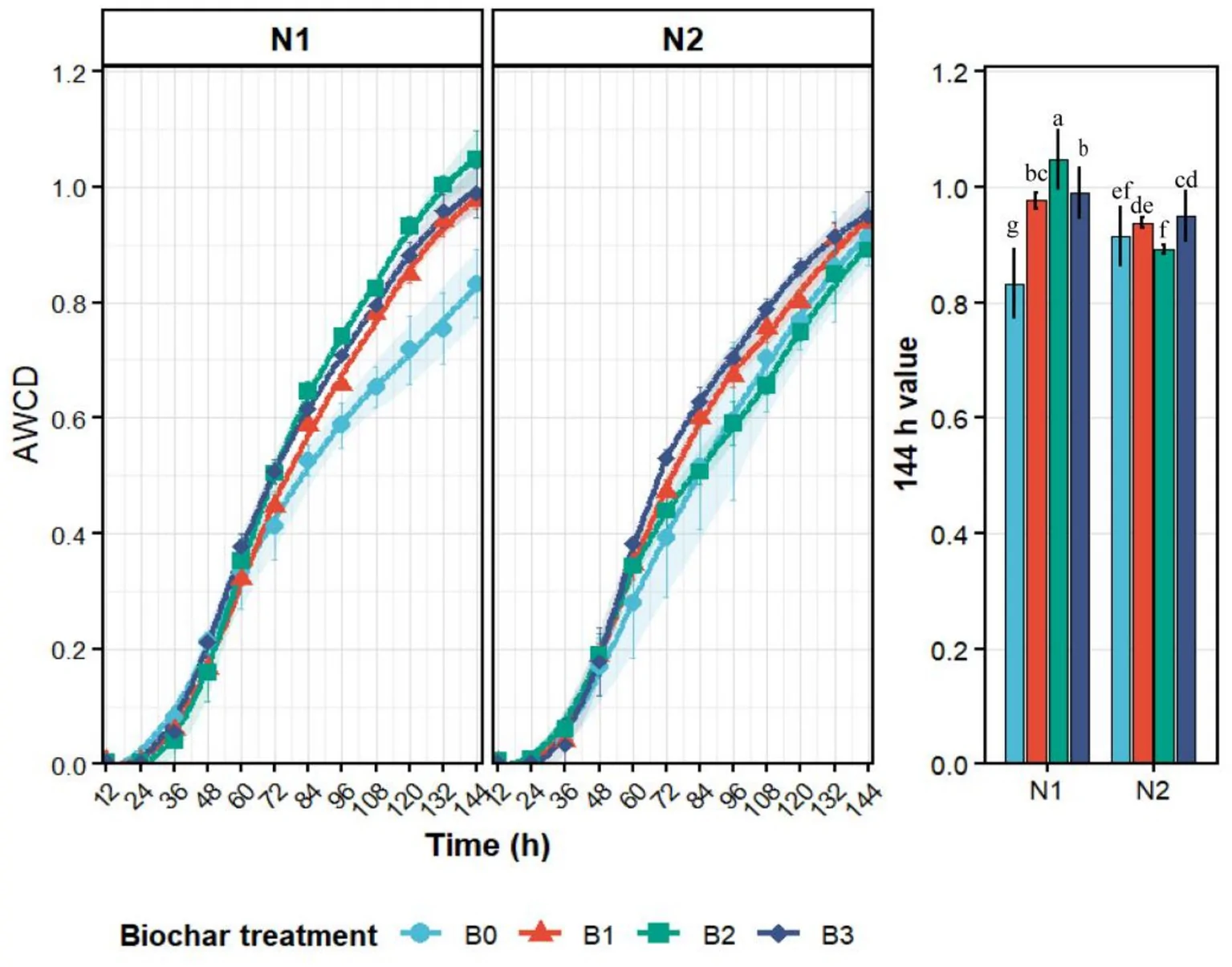
AWCD values of carbon sources under different treatments.

AWCD reveals the extent to which soil microorganisms can use the carbon sources for their metabolic activities. A higher AWCD value indicate higher microbial activity in soil. As shown in Figure 1, carbon source utilization by soil microorganisms increased with the rising cultivation time, exhibiting an S-shaped trend. In the beginning of cultivation (0–36 h), weak microbial activity was observed, with a slow rise in AWCD. From 36 to 120 h, microbial activity increased, with a rapid rise in carbon source utilization. After 120 h, the activity stabilized, and the carbon utilization efficiency of microorganisms enhanced in all treatment groups compared to N1B0 group. The strongest microbial activity was observed in N1B2 group, including moderate biochar application with conventional nitrogen fertilization.

### 3.2 Effect of biochar dose on diversity indices

The functional diversity indices (Simpson, Shannon, and Mcintosh) of microbes in the farmland soil were calculated using the OD at 590 nm after 120 h of cultivation. These indices reflected the distribution and evenness of carbon-substrate utilization capabilities among the culturable microorganisms (Table 1). Biochar addition altered the Shannon and Mcintosh indices of soil microorganisms, with notable variations among the treatments (*p* < 0.05). When both substances were added to soil, the Mcintosh index showed a substantial rise, but declined with a decrease in nitrogen fertilizer application. Among the treatments, N1B2 treatment led to the highest Mcintosh index (6.54), which was 20% higher compared to N1B0 group. The Shannon index rose with the increasing rates of nitrogen fertilizer and biochar addition. Overall, these additives exhibited an interactive effect on the functional diversity of soil microbes in this study.

**Table 1:** Functional diversity indices of microbes in soil under different treatments.

Treatment	AWCD	Simpson index (D)	Shannon index (H)	Mcintosh index (U)
N1B0	0.72 ± 0.02f	0.94 ± 0.00a	2.96 ± 0.01f	5.45 ± 0.14d
N1B1	0.85 ± 0.01c	0.95 ± 0.00a	3.13 ± 0.01a	5.83 ± 0.03c
N1B2	0.93 ± 0.01a	0.95 ± 0.00a	3.11 ± 0.00b	6.54 ± 0.06a
N1B3	0.88 ± 0.01b	0.95 ± 0.00a	3.13 ± 0.00a	6.09 ± 0.07b
N2B0	0.77 ± 0.01e	0.94 ± 0.00a	3.01 ± 0.01e	5.86 ± 0.08c
N2B1	0.80 ± 0.01d	0.94 ± 0.00a	3.01 ± 0.00de	6.08 ± 0.04b
N2B2	0.75 ± 0.01e	0.95 ± 0.00a	3.03 ± 0.01d	5.41 ± 0.04d
N2B3	0.86 ± 0.01bc	0.95 ± 0.00a	3.07 ± 0.01c	5.95 ± 0.03bc
B	ns	ns	ns	ns
N	*	ns	ns	ns
B × N	**	**	**	**

Values are treatment means based on three independent field replicates (*n* = 3). Different lowercase letters within the same column indicate significant differences among treatment combinations at *p* < 0.05 according to Duncan’s multiple range test following two-way ANOVA. ns, *, and ** indicate *p* ≥ 0.05, *p* < 0.05, and *p* < 0.01, respectively.

### 3.3 Effect of biochar application on carbon source utilization capacity of soil microbes

The Biolog-Eco biochips contain a total of 31 carbon sources, including 6 amino acids, 8 acids, 7 sugars, 4 esters, 3 alcohols, and 3 amines. Using the OD values recorded after 120 h of incubation, the carbon consumption ability of microorganisms were determined for 6 kinds of carbon sources.

**Figure 2:**
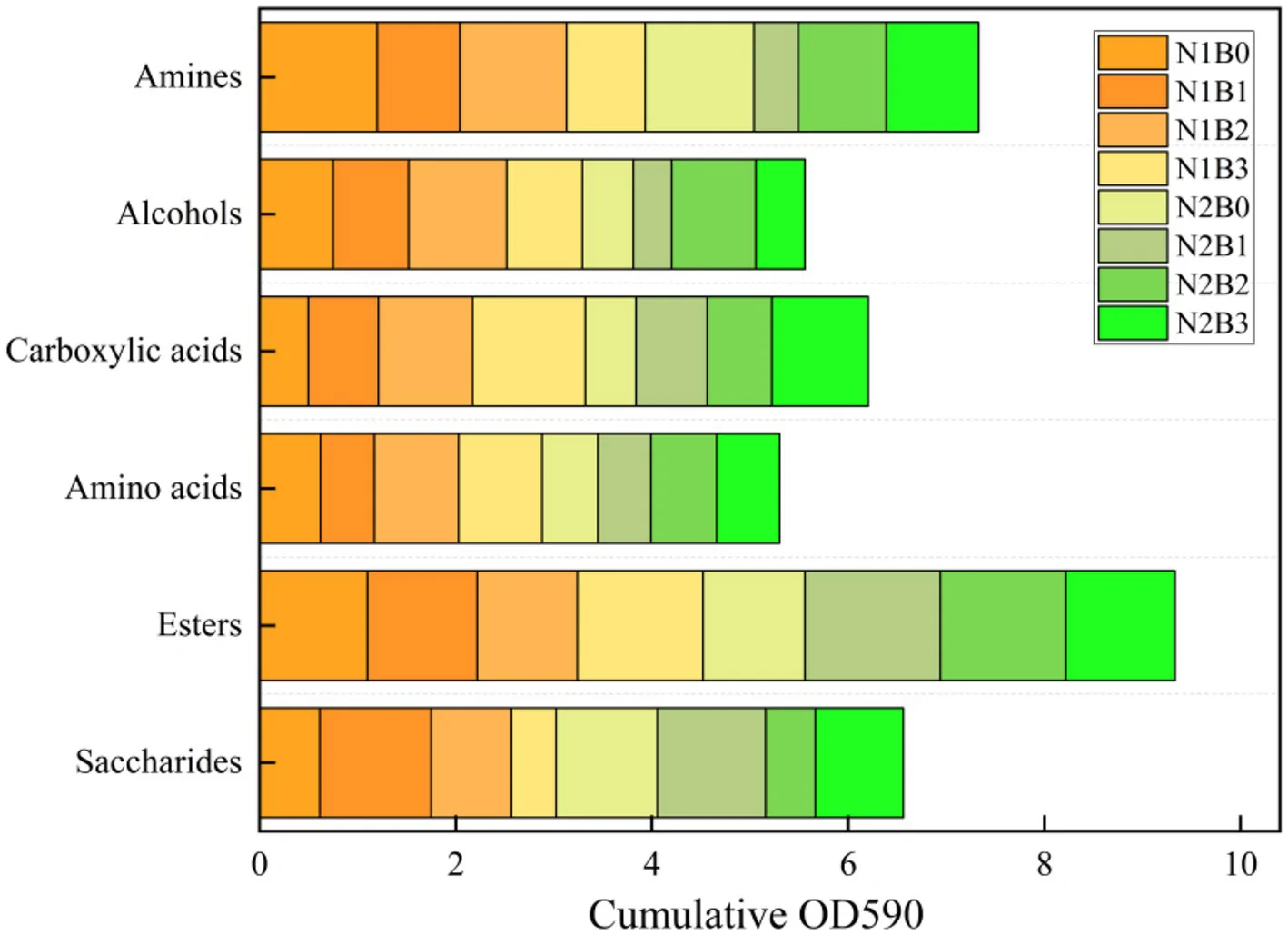
Accumulation of different carbon sources.

**Figure 3:**
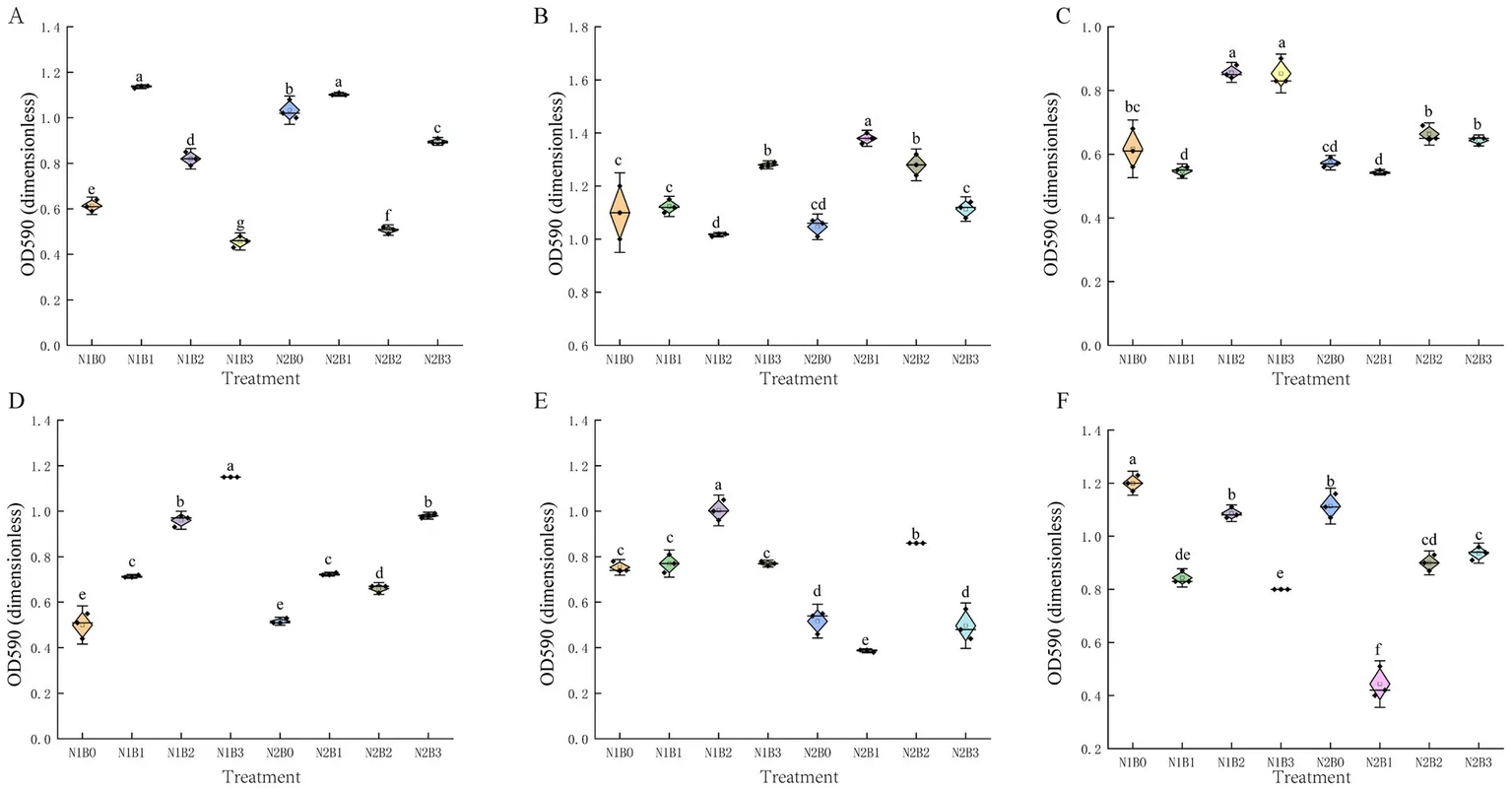
Variations in the ability of soil microbes to use different sources of carbon under different treatments. **(A)** Saccharides, **(B)** Esters, **(C)** Amino acids, **(D)** Carboxylic acids, **(E)** Alcohols, **(F)** Amines.

Figure 2 showed the highest mean utilization per substrate for ester-group substrates, followed by amines. Overall, amino acid utilization remained low under all treatments. Further assessment revealed varying capacities of soil microbes to utilize carbon sources under different treatments (Figures 3A–F). Compared to N1B0, the coupled application of both substances boosted the carbon source utilization capacity of soil microorganisms. The highest sugar utilization was observed under N1B1 treatment, including conventional nitrogen fertilization with low-dose biochar application, with substantial variations between the groups (*p* < 0.05) (Figure 3A). Overall, all treatment groups showed high consumption of esters by soil microorganisms (Figure 3B). Peak ester utilization (OD590: 1.38) was observed under N2B1 treatment, with substantial variations between the groups (*p* < 0.05). The acid utilization capacity of soil microorganisms varied with the changes in the rate of biochar application across treatments (Figure 3D). Under N1B2 and N1B3 treatments, this capacity decreased with the decline in nitrogen fertilizer application rate. The highest OD590 value (1.15) was witnessed in N1B3 group. Compared to N1B0, amine utilization by soil microbes declined across all treatments, with noticeable variations between the groups (*p* < 0.05). The lowest amine utilization was noticed under N2B1 treatment (Figure 3F).

The data obtained from colorimetric analysis after 120 h of cultivation were subjected to principal component analysis (PCA). The first principal component (PC1) and second principal component (PC2) contributed 44.4 and 32.6% of the variance, respectively, with combined contribution of 77% (Table 2). PC1 encompassed amino acids and alcohol carbon sources, while PC2 included esters and amines (Table 3).

**Table 2:** Results of PCA analysis.

Ingredients	Initial eigenvalues		
	Eigenvalue	Percentage of variance (%)	Cumulative (%)
1	2.66	44.39	44.39
2	1.95	32.59	76.98

**Table 3:** Load matrix of two principal components.

Types of carbon sources	Ingredients	
	PC1	PC2
Saccharide	−0.44	−0.20
Esters	−0.22	0.59
Amino acids	0.54	0.25
Carboxylic acids	0.28	0.48
Alcohols	0.51	−0.08
Amines	0.34	−0.56

**Figure 4:**
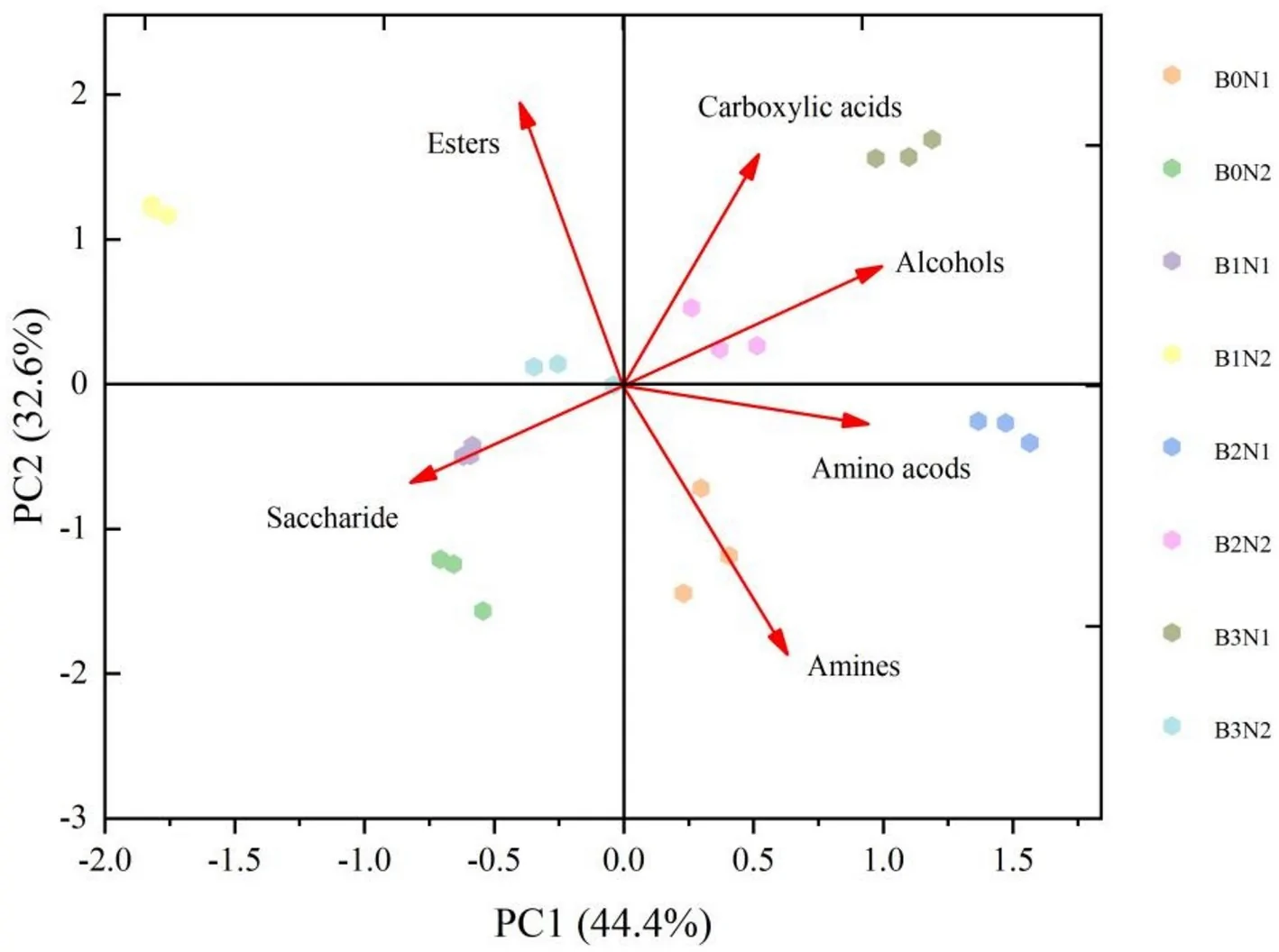
PCA plot showing carbon source utilization by soil microbes under different treatments.

The treated soil samples placed in the same quadrant of PCA plot (PC1 on x-axis and PC2 on y-axis) exhibited similar carbon-utilization functional profiles, while those in different quadrants had varying profiles. Figure 4 illustrates the variations in carbon source consumption scores of different treatment groups distributed across all quadrants. These findings indicate that simultaneous addition of both additives altered the carbon-metabolic functional composition of the culturable soil microbial fraction.

### 3.4 Impact of biochar dose on cumulative CO_2_ emissions from soil

**Figure 5:**
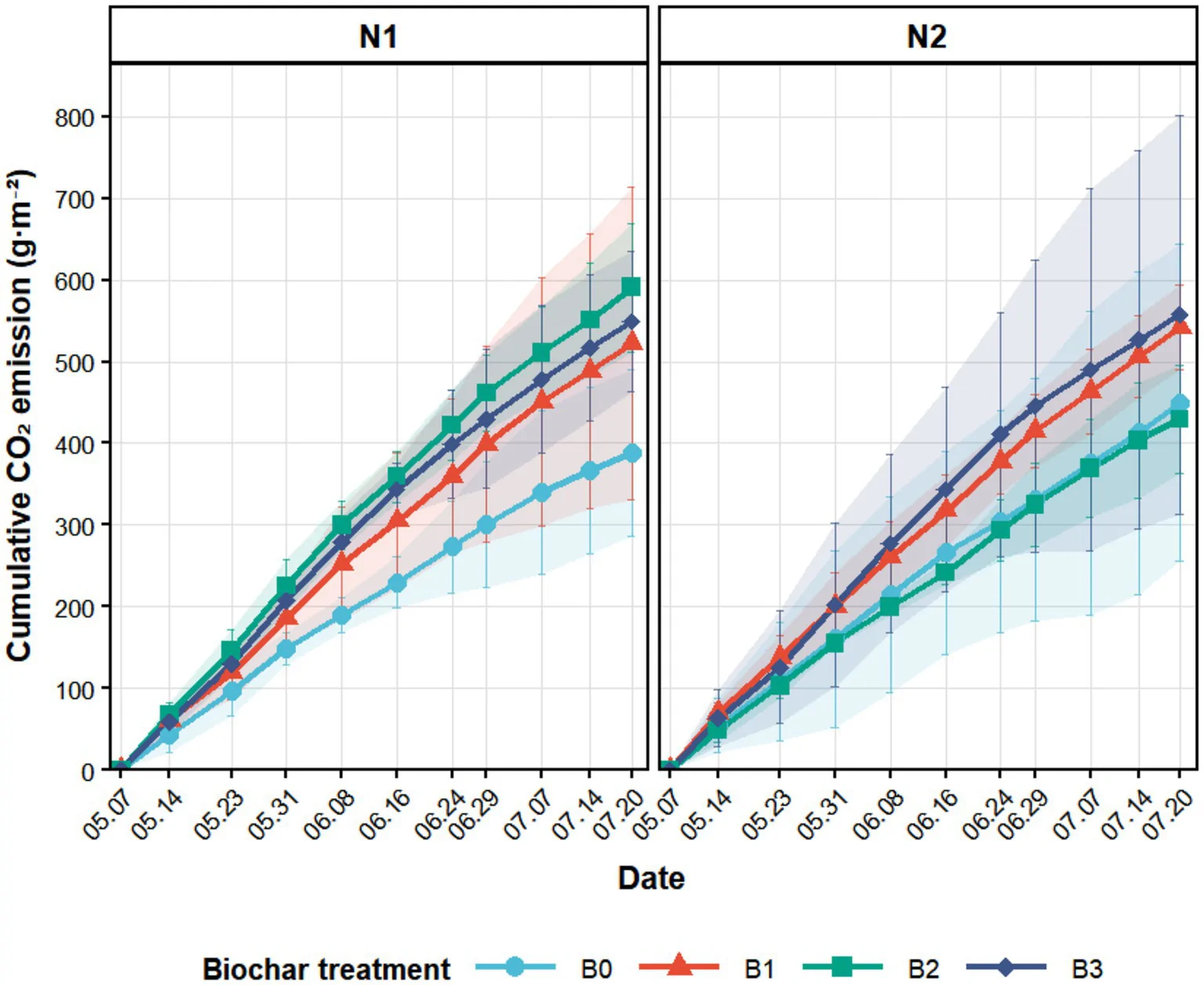
Cumulative release of CO_2_ from soil.

Figure 5 shows that biochar treatment caused a swift rise in cumulative CO_2_ emissions during the initial fertility stage of wheat (0–48 days), followed by a relatively slow rise in the later stage (48–84 days). Compared to N1 groups, N2 treatments showed a remarkable surge in collective release of CO_2_. Also, all treatments, except for N1B0 and N2B0, led to higher cumulative CO_2_ emissions compared to N1B0 (Figure 5). In the N1 groups, cumulative CO_2_ emissions raised with the rising biochar application rates. The N1B3, N1B2, and N1B1 groups showed 41.2% (*p* < 0.05), 51.9% (*p* < 0.05), and 34.3% higher CO_2_ emissions, respectively, than the N1B0 group. On the other hand, N2 groups showed the trends comparable to N1 groups. The lowest cumulative CO_2_ emissions (429.45 g·m^−2^) was witnessed in N2B2 group, which was 4.4% lower than that under N2B0 treatment.

### 3.5 Impact of nitrogen fertilizer and biochar doses on soil enzyme activity

**Figure 6:**
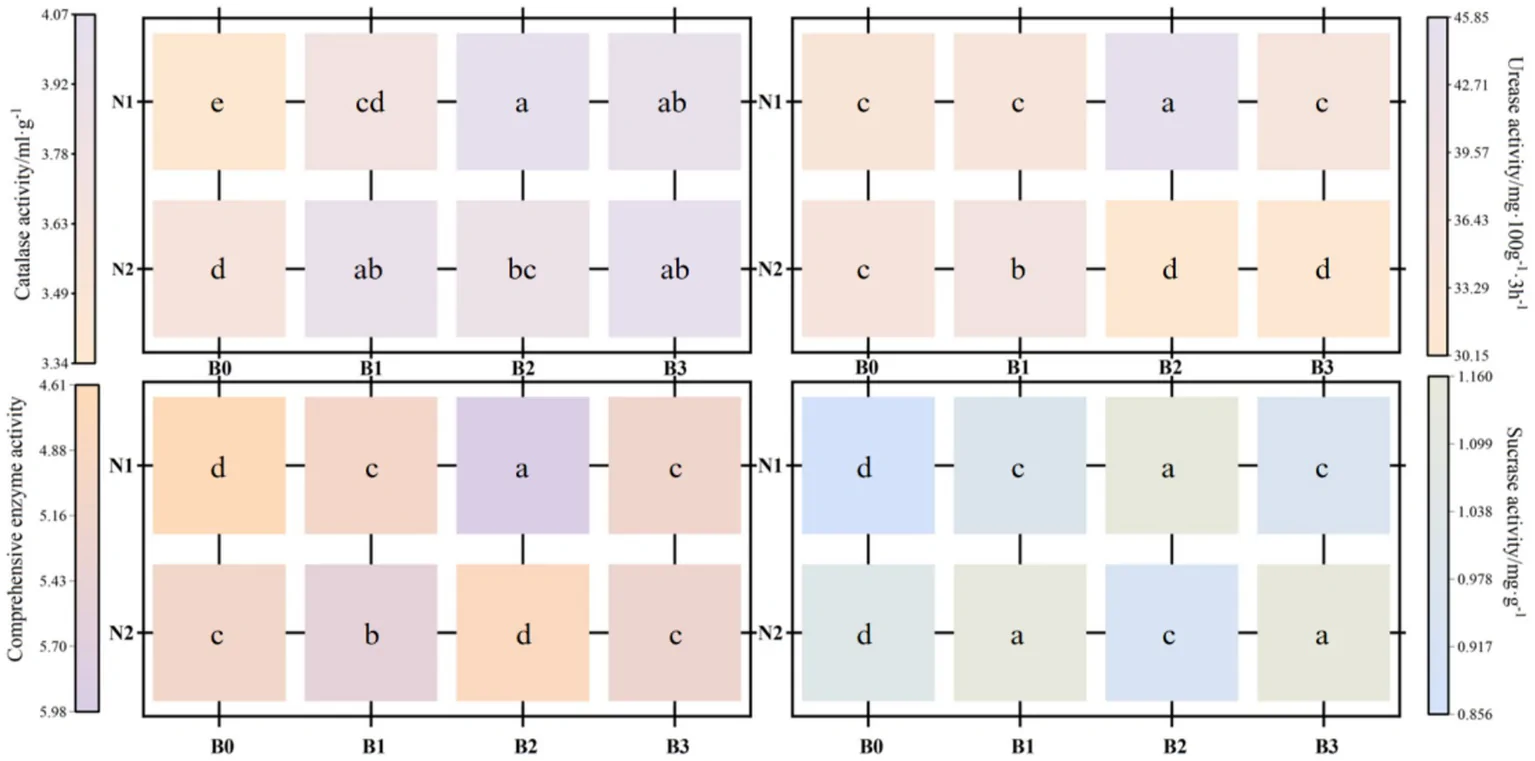
Effects of nitrogen application rates (N1, N2) and biochar addition levels (B0–B3) on catalase activity, urease activity, comprehensive enzyme activity, and sucrase activity in soil. Different lowercase letters denote significant differences between treatments at *p* < 0.05. Each panel is accompanied by an independent color gradient scale corresponding to the measured values of enzyme activity.

Urease activity differed significantly among the treatments (*p* < 0.05; Figure 6). In the absence of biochar, urease activity was 33.7% higher under reduced nitrogen treatment (N2B0) than under conventional nitrogen treatment (N1B0). Biochar application significantly affected urease activity, although the response varied with nitrogen application rate. Under conventional nitrogen application, urease activity initially increased and then decreased with increasing biochar application rate. The highest urease activity (45.84 mg·100 g^−1^·3 h^−1^) was observed under N1B2, representing an increase of 60.5% compared with N1B0 (*p* < 0.05). Under reduced nitrogen application, urease activity was 15.2 and 13.3% lower under N2B2 and N2B3, respectively, than under N2B0 (*p* < 0.05).

Notable inter-group variations (*p* < 0.05) in sucrase activity were observed. The N2B0 group exhibited 17.2% higher activity than N1B0. Coupled treatment of soil with biochar and nitrogen fertilizer resulted in higher sucrase activity. In the N1 groups, sucrase activity first surged and then declined with soaring rates of biochar application. The N1B2 group exhibited highest sucrase activity (1.14 m·g^−1^). In the N2 groups, sucrase activity initially declined and then increased with soaring biochar levels. Compared to N2B0, N2B3 and N2B1 groups showed 9.32 and 11.89% higher sucrase activity, correspondingly (*p* < 0.05).

Catalase activity also differed notably among the treatment groups. N2B0 group showed 7.8% higher activity of catalase than N1B0. In the N1 groups, catalase activity surged and later declined with rising dose of biochar. N1B2 treatment led to greatest catalase activity (4.97 mL·g^−1^). In the N2 groups, catalase activity declined and then increased with rising biochar application rates. N2B3, N2B2, and N2B1 groups showed increases of 11.7, 7.8, and 10.2% in catalase activity, respectively, than that under N2B0 treatment.

The geometric mean activity of sucrase, catalase, and urease under different treatments was determined as the total enzyme activity in the farmland. All groups showed notable increase in enzyme activity than N1B0 (*p* < 0.05, Figure 6). The N1 groups displayed elevated geometric mean activity, with the N1B2 group showing the peak mean activity (5.97).

## 4 Discussion

Microbial functional diversity offers an excellent assessment of soil quality. Regulating the metabolic activity of soil microbes can alleviate the decline in soil fertility and achieve sustainable development (Pei et al., 2025). However, the impact of biochar on soil microbial metabolic function remains uncertain (Wang et al., 2025a; Wang et al., 2025b; Wu et al., 2021). In this study, Biolog-ECO ecological discs were employed to show that, compared to N1B0, joint application of biochar and nitrogen fertilizer caused notable increase in AWCD, H, and U values, indicating marked increase in microbial activity. Under reduced application of nitrogen fertilizer, the Shannon index improved with the rising rates of biochar addition. This implies that biochar and nitrogen fertilizer, when applied together, exhibited synergistic effects and improved the metabolic activity of soil microorganisms. This finding is consistent with the results reported by Liu H. et al. (2025). According to a previous study, biochar and nitrogen fertilizer modulate soil C/N ratios (Dey and Mavi, 2021), providing ample nutrients to soil microorganisms, while the porosity and highly aromatic structure of biochar influence the structure and physical properties of soil. Large aggregates of soil form substantial voids between the aggregates, enhancing oxygen availability for aerobic microorganisms and stimulating microbial activity (Dai X. et al., 2021; Dai Z. et al., 2021; Yang C. et al., 2022; Yang Y. et al., 2022). Furthermore, biochar had excellent capacity to adsorb water and nutrients. The trace nutrients in biochar create favorable growth environment for microorganisms, thereby improving microbial activity in the soil (Hu et al., 2024; Ramezanzadeh et al., 2023). Compared to N2 groups (reduced nitrogen fertilizer plus biochar), N1 groups with conventional nitrogen fertilizer and biochar application showed higher microbial activity. This divergence may be attributed to reduced application of nitrogen fertilizer, which directly reduces available nitrogen in the soil. These changes restrain the growth and reproduction of aerobic autotrophic nitrogen-fixing bacteria and ammonifying bacteria (Dai X. et al., 2021; Dai Z. et al., 2021), which further affects microbial activity in soil (Shi et al., 2023).

Further analysis showed that biochar caused notable rise in the consumption of various carbon sources (amino acids, lipids, and acids) by soil microorganisms. After treatment with nitrogen fertilizer or biochar alone, the key sources of carbon consumed by microorganisms were mostly similar to those in the control. When these additives were applied together, microbial capacity to use amines, amino acids, and acids as carbon sources declined with reducing levels of nitrogen fertilizer. This indicates that lower rate of nitrogen fertilizer application declined the carbon metabolic activity of soil microorganisms (Chen et al., 2022). In our previous research, nitrogen fertilization with high dose of biochar had no effects on soil microorganisms (Yang C. et al., 2025; Yang W. et al., 2025). On the contrary, changes in the primary sources of carbon consumed by soil microorganisms were observed in this study when biochar and nitrogen fertilizer applied together. The variations relied on the dose of biochar. This indicates that simultaneous application of these substances produces an interactive influence, altering carbon-metabolic functional profiles in the soil. Within a suitable range, rising biochar levels caused notable surge in microbial activity. This may be because nitrogen fertilizer offset the relatively low level of nutrients in biochar. Biochar’s porous structure and adsorption capacity may influence nitrogen retention and availability, which could in turn affect microbial nitrogen acquisition. However, direct measurements of gaseous nitrogen losses (e.g., N_2_O, NH_3_) were not conducted in this study, and thus these mechanisms remain speculative. (Borchard et al., 2019; Xia et al., 2020; Chen et al., 2025). These balancing interactions likely trigger rapid proliferation of some microbial taxa after addition of both substances, thereby modifying carbon-utilization functional profiles. However, at high application rates, biochar may impose suppressive effects on microbial activity, potentially due to elevated pH, salinity, or other physicochemical constraints associated with the biochar material. The specific inhibitory factors were not identified in this study, and further research is needed to elucidate the mechanisms underlying the threshold response observed at higher biochar rates (Si et al., 2024; Liu C. et al., 2025).

Through soil respiration, organisms and the below-ground plant parts of plants in soil generate CO₂, including root respiration and heterotrophic microbial respiration (Bond-Lamberty et al., 2024; Wang et al., 2025a; Wang et al., 2025b). In this study, the sum of these two types of respirations represented soil respiration. Previous studies on biochar’s influence on CO₂ emissions from soil have reported controversial findings. Most studies reported the CO₂ sequestration ability of biochar, which can reduce CO₂ emissions (Lehmann et al., 2021; Wang et al., 2023). However, some studies have demonstrated increase in CO₂ emissions after biochar addition, with improved soil properties (Babichuk et al., 2025). In this study, different application rates of additives led to varying respiration rates. Biochar addition with conventional nitrogen fertilization resulted in increased cumulative CO₂ emissions from soil. Soil active organic carbon (SAC) is the main source of carbon for the growth and metabolic activities of microorganisms. It swiftly undergoes decomposition and mineralization in soil while being susceptible to conversion and migration. Thus, SAC content can directly affect the CO₂ emissions from soil (Wang et al., 2026; Xu et al., 2025; Wen et al., 2025). Biochar addition improves the porosity (Wong and Ogbonnaya, 2021; Jia et al., 2024; Leng et al., 2021) and moisture level (Fakhar et al., 2025; Adekiya et al., 2025; Liang et al., 2025) in the soil, boosting mineralization and enhancing the content of easily mineralizable soluble organic matter (Ameloot et al., 2015). Furthermore, biochar causes an increase in soil pH (Zhang et al., 2025), improves the abundance of electronegative functional groups on humic colloids and stimulates base cations (Gui et al., 2021). These changes accelerate the transformation of native organic matter into soluble forms, thereby promoting mineralization (Shi et al., 2025). Furthermore, the organic matter content is mainly stored in soil microaggregates (<0.25 mm). Soil microaggregates have a higher C/N ratio than macroaggregates. These microaggregates suppress the disintegration of organic matter. Biochar may improve the disintegration of organic matter by improvingmacroaggregates-to-microaggregates ratio in soil, resulting in higher CO₂ emissions (Zhang et al., 2021; Yang C. et al., 2022; Yang Y. et al., 2022). In our previous research, moderate dose of biochar improved the stability of soil aggregates (Yang et al., 2024), physically sequestering organic carbon and inhibiting its enzymatic and microbial decomposition (Yang C. et al., 2025; Yang W. et al., 2025; Li et al., 2018). However, at higher application rates, vast specific surface area and strong adsorption capacity of biochar boosted the activity of microbes (Kayoumu et al., 2025), thereby supporting microbial respiration in the soil (Parasar and Agarwala, 2025; Enebe et al., 2025). In this study, under coupled treatment of biochar and reduced nitrogen fertilizer, cumulative emissions of CO₂ first declined and then surged with rising levels of biochar, demonstrating the impact of biochar on soil respiration. In N2B2 group with moderate biochar level, cumulative release of CO₂ from soil was lower than those under N2B0. This observation aligns with the findings of Wang et al. (2020). In some studies, biochar did not exert any impact on soil respiration (Vannini et al., 2025; Liu et al., 2016). It is important to note that the cumulative CO₂ emissions reported here represent soil CO₂ efflux only, not the net carbon balance of the system. Biochar adds stable carbon to the soil, and its net effect on greenhouse gas balance depends on the trade-off between biochar-derived carbon sequestration and any stimulated native soil organic matter mineralization (i.e., positive priming). In the present study, only soil CO₂ efflux was measured; therefore, we refrain from claiming net carbon sequestration. Our results instead indicate that, under most treatment combinations tested, biochar addition stimulated soil CO₂ efflux, with the exception of the N2B2 treatment, which showed a modest reduction relative to N2B0.

Soil enzyme activities and total soil respiration both responded to nitrogen and biochar application. However, the quantitative relationship between enzyme activities and soil CO₂ emissions was not directly evaluated in this study. Therefore, the observed changes in enzyme activities should not be interpreted as direct causes of the changes in soil respiration. Biochar improves the levels of organic substances and nutrients in soil, supporting microbial growth and reproduction, which leads to higher microbial diversity and abundance, as well as faster enzymatic reactions. Moreover, biochar, with its porous structure and large specific surface area, provides favorable sites for microbial colonization (Jaafar et al., 2015). The observed increases in enzyme activities may reflect enhanced microbial metabolic activity or shifts in the functional composition of the culturable microbial community. However, we did not directly measure microbial biomass carbon or cell abundance in this study, and therefore the underlying cause of elevated enzyme activities cannot be definitively attributed to biomass increases (Song et al., 2022). High-dose biochar application notably improves protease activity (Imparato et al., 2016) than low-dose applications. At low dosage of biochar, microbial activity increase may be attributed to the carbon source and the beneficial habitat provided by porous biochar structure. However, at higher addition rates, extremely high pH of biochar can suppress enzyme activity, thus impeding microbial respiration (Chen et al., 2019). In this study, addition of both substances set off the drawbacks of separate treatments with nitrogen fertilization (carbon shortage) and biochar (nitrogen shortage). It accelerates the enzymatic reactions, improves microbial activity, and promotes the activities of catalase, sucrase, and urease (Khan et al., 2021; Jiang et al., 2021). In this study, when biochar application was coupled with conventional nitrogen fertilization, enzyme activity rose and subsequently declined with soaring biochar levels, demonstrating the threshold impact of biochar. At high application rates, biochar may impose suppressive effects on enzyme activity, potentially due to elevated pH, salinity, or other physicochemical constraints. However, the specific inhibitory factors were not identified in this study, and further research is needed to elucidate the mechanisms underlying the threshold response observed at higher biochar rates.

It should be noted that the BIOLOG EcoPlate method employed in this study captures the carbon-metabolic functional diversity of the culturable soil microbial fraction, rather than taxonomic community composition. While the present analysis is based on data from the 2021 growing season, this experiment is part of an ongoing long-term positioned trial in which complementary 16S rRNA amplicon sequencing has been conducted in subsequent years to characterize soil bacterial and fungal community structure. Those findings, published separately, will further elucidate the taxonomic-level microbial responses to biochar and nitrogen fertilization in this agroecosystem.

## 5 Conclusion

Biochar treatments can enhance soil conditions by influencing the activity of key enzymes in soil. The influence of biochar on CO₂ release from soil relies on the application rate. Short-term application of biochar in the farmland in the Northern Xinjiang Irrigation District can sustain or improve the crop yields but may not reduce CO₂ release completely. Under the experimental conditions, the N2B2 treatment (reduced nitrogen + moderate biochar) showed the lowest cumulative CO₂ emissions among all treatments. However, net carbon sequestration potential requires further assessment through long-term soil organic carbon stock monitoring, as the present study measured only soil CO₂ efflux rather than full carbon balance. Nevertheless, N2B2 may serve as a promising fertilization strategy for mitigating CO₂ efflux in wheat fields of the North Xinjiang Irrigation District, pending validation through long-term observations.

## Data Availability

The original contributions presented in the study are included in the article/supplementary material, further inquiries can be directed to the corresponding author.
